# COVID-related psychological distress fully mediates the association from social impact to sleep disturbance among patients with chronic schizophrenia

**DOI:** 10.1038/s41598-021-96022-2

**Published:** 2021-08-16

**Authors:** Dian-Jeng Li, Li-Shiu Chou, Frank Huang-Chih Chou, Su-Ting Hsu, Kuan-Ying Hsieh, Hui-Ching Wu, Wei-Tsung Kao, Guei-Ging Lin, Wei-Jen Chen, Joh-Jong Huang

**Affiliations:** 1 Kaohsiung Municipal Kai-Syuan Psychiatric Hospital, No. 130, Kaisyuan 2nd Rd., Lingya District, Kaohsiung, 802211 Taiwan; 2 Department of Nursing, Meiho University, Pingtung, Taiwan; 3 Graduate Institute of Medicine, College of Medicine, Kaohsiung Medical University, Kaohsiung, Taiwan; 4 Department of Social Work, National Taiwan University, Taipei, Taiwan; 5 Department of Sports, Health and Leisure and Graduate Institute of Sports, Health and Leisure, Cheng Shiu University, Kaohsiung, Taiwan; 6 Graduate Institute of Counseling Psychology and Rehabilitation Counseling, National Kaohsiung Normal University, Kaohsiung, Taiwan; 7 Department of Family Medicine, Kaohsiung Medical University Hospital, Kaohsiung City, Taiwan; 8 Department of Health, Kaohsiung City Government, No.132-1, Kaisyuan 2nd Rd., Lingya District, Kaohsiung, 802212 Taiwan

**Keywords:** Social behaviour, Stress and resilience, Epidemiology, Risk factors, Schizophrenia

## Abstract

The aims of the current study were to identify factors associated with sleep disturbance and Coronavirus disease-19 related psychological distress (CPD), and to develop a conceptual model to verify the mediating effect of CPD on the association between social impact and sleep disturbance. This study recruited patients with schizophrenia. Factors associated with the level of sleep disturbance and CPD were identified using univariate linear regression, and further selected into a stepwise multivariate linear regression model. Using structural equation modeling, a mediation model was developed to test the mediating effect of CPD on the association between social impact and sleep disturbance. After estimating with the stepwise and bootstrap regression, higher levels of CPD were associated with higher levels of social anxiety and subjects without a regular diet. Sleep disturbance was associated with a higher level of social anxiety, a history of psychological trauma, chronic disease, and those who did not smoke. The final model confirmed the mediating effects of CPD; whereas, the direct effect from social impact to sleep disturbance did not reach statistical significance. The current study manifests the crucial role of CPD on the association between social impact and sleep disturbance, and timely intervention for CPD is warranted.

## Introduction

### Upcoming threat of COVID-19 on patients with schizophrenia

Coronavirus disease-19 (COVID-19) has rapidly spread globally with an enormous number of deaths that continues to climb. It can also have a multi-dimensional effect on patients with schizophrenia. Regarding the significant risks of pneumonia among patients with schizophrenia^1^, it can also compromise immune function, increasing the risk of developing a respiratory infection^2^. In addition, up to 70% of schizophrenia patients have one or more medical comorbidities^3^, and these clinical conditions can increase risk of infection^4^ and mortality rates for COVID-19^5^. For instance, smoking is prevalent in 60% of patients with schizophrenia^6^ and therefore increases the risk of disease progression for COVID-19 through its effects on immune responsiveness^7^. Therefore, patients with schizophrenia may fit in the high-risk infection group for COVID-19. Another threat is the burden of availability to the medical service. Individuals with psychotic disorders have significantly lower rates of registration with primary care physicians and they can have greater difficulties in assessing adequate care compared with those without mental disorders^8^. Furthermore, stigmatization of schizophrenia may also hinder patients from receiving a proper assessment or treatment within the medical services^9^.

### COVID-related psychological burden among patients with schizophrenia

Psychological distress caused by COVID-19 resulted in poor outcome of mental health. It revealed that more than one-fourth of the publics in China suffered poor sleep quality during the COVID-19 outbreak^10^. Another study with large Spanish sample manifested the problem of sleep disturbance during pandemic, where it was associated with depression, anxiety, and distress^11^. Moreover, this psychological burden caused by COVID-19 has also been reported by patients with mental illness, including schizophrenia. A cross-sectional study demonstrated that patients with a pre-existing psychiatric disorder had predominant distress along with the presence of loneliness and depressive symptoms^12^. Another study revealed that patients with severe mental illness (psychotic or bipolar disorder) responded to the COVID-19 pandemic and the lockdown restrictions with significantly higher levels of anxiety than the general population^13^. In addition, patients with schizophrenia have been encountered who have a massive psychological burden due to COVID-19. Patients with schizophrenia who were under isolation due to suspected COVID-19 showed significantly higher levels of stress, depression and anxiety, as well as poorer sleep quality in comparison with patients in the non-suspected group^14^. It is possible that COVID-19 infection could exacerbate the symptoms of schizophrenia, as coronaviruses are associated with the emergence of psychotic symptoms through immune-related mechanisms^15^. Furthermore, treatment for COVID-19 is also associated with psychological burden, such as the distress of medical isolation and psychosis secondary to steroids and other interventions^16^. Although the available studies are insufficient, increased attention should be paid to the psychological distress of schizophrenia patients, due to the undesirable impacts of COVID-19.

### Social impact of COVID-19 on patients with schizophrenia

Social distancing and related policies, whereby authorities have discouraged and imposed restrictions to reduce physical proximity, may interfere with social interactions during the COVID-19 outbreak. In Taiwan, people suffered from social impacts of COVID-19 due to policies of infection control, which was announced by the Taiwan Centers for Disease Control (Taiwan CDC). At the early June of 2020, around 450 of cases were infected with COVID-19^17^. Besides strict broader control, the Taiwan CDC also declared a series of strategies for inpatients, including forced wearing a mask, taking body temperatures, and limitation for visitors in hospital.

Changes in social interactions can trigger harmful effects on the public’s mental health, such as sleep disturbances and suicidal ideas^18^. As for individuals with schizophrenia, social distancing practices have a particularly negative impact. Often due to the disease’s negative symptoms, patients with schizophrenia have smaller and poorer-quality social networks than the general population^19^. Thus, social support and associated rehabilitation programs can be helpful for recovery^20^. Distal social supports, including casual contacts at pharmacies, grocery stores, restaurants/cafes and other stores, can enhance community integration for patients with schizophrenia^21^. The above interventions will be easily disrupted by social distancing and related policies, resulting in a poorer prognosis for the disease. In addition, social isolation among individuals with schizophrenia is a risk factor for suicide^22^. As a result, further exploration of the social impact of COVID-19 on this population is warranted to identify the effects it has on disease progression.

### Aim of the current study

The massive impact of COVID-19 on patients with schizophrenia has only been preliminarily explored, further studies regarding the risk factors associated with the severity of multi-dimensional impacts are urgently needed. Furthermore, the interaction between these impacts are as yet unidentified. Therefore, a cross-sectional study was conducted to comprehensively investigate the associations between the impacts of COVID-19 and related predictors. Two of the aims of the current study were as follows: (1) several factors, especially the social impacts of COVID-19, were analyzed to identify if they were associated with COVID-related psychological distress and sleep disturbance; (2) if a significant association was found, a conceptual model was developed to examine the interactions among them. We hypothesized that COVID-related psychological distress mediates the association between social impact and sleep disturbance.

## Results

### Summary of demographic analysis and reliability of the questionnaires

In total, 392 of the subjects fitted the inclusion criteria. Those who replied to the forms with missing values (n = 11) were excluded, and the remaining 381 subjects were entered into the analysis. The source distribution for the participants was as follows: chronic ward (n = 76; 19.95%), day care ward (n = 37; 9.71%), half-way house (n = 76; 19.95%), nursing home (n = 81; 21.26%), and community rehabilitation center (n = 111; 29.13%). A total of 149 females and 232 males participated in this study, and the mean age of the participants was 50.43 ± 11.34 years. Among the dependent variables, the mean scores of sleep disturbance and COVID-19 related psychological distress were 5.67 ± 2.28 and 5.28 ± 2.56, respectively. The reliability estimated by Cronbach’s α for scales of sleep disturbance, COVID-19 related psychological distress, social distance, social anxiety, social information, and social adaptation were 0.78, 0.86, 0.85, 0.62, 0.66 and 0.68, respectively (Supplementary Tables S1). All of the values were above 0.5, indicating an acceptable range^23^. In addition, the validity test with total variance explained revealed the range from 47.7 to 61.8% (Supplementary Tables S1). All of them reached or were close to the requirement of the threshold at 50 percent of the total variance^24^. The remaining summaries of characteristics for all individuals are listed in Table 1. Furthermore, the distribution of marital status, history of psychological trauma, and chronic medical disease are presented in Supplementary Tables S2–S4.

**Table 1:** Predictors for level of sleep disturbance examined by univariate and stepwise multivariate linear regression (N = 381).

Predictors			Univariate regression				Stepwise multivariate regression			
	Mean	SD	β	t	95% CI	p	β	t	95% CI	p
Age (years)	50.43	11.34	−0.09	− 1.72	− 0.04, 0.002	0.086	–	–	–	**–**
Education level (years)	12.02	3.20	0.09	1.71	− 0.01, 0.14	0.088	–	–	–	**–**
Social distance	9.31	4.13	0.11	2.17	0.01, 0.12	**0.031**	− 0.05	− 1.01	–	0.316^a^
Social anxiety	3.48	1.75	0.36	7.50	0.35, 0.59	** < 0.001**	0.30	6.17	0.27, 0.52	** < 0.001**
Social information	4.24	1.90	0.18	3.49	0.09, 0.33	**0.001**	− 0.04	− 0.74	–	0.435^a^
Social adaptation	4.37	2.23	0.14	2.73	0.04, 0.25	**0.007**	− 0.04	− 0.84	–	0.403^a^

	*n*	%	β	t	95% CI	*p*	β	t	95% CI	*p*
**Sex**										
Male	232	60.9	Ref	–	–	–	Ref	–	–	–
Female	149	39.1	0.16	3.06	0.26, 1.19	**0.002**	0.07	1.42	–	0.155^a^
**Occupational status**										
Employment	48	12.6	Ref	–	–	–	–	–	–	–
Unemployment	333	87.4	− 0.02	− 0.46	− 0.86, 0.53	0.649	–	–	–	**–**
**Marital status**										
Without partner	342	89.8	Ref	–	**–**	–	–	–	***–***	*–*
With partner	39	10.2	− 0.06	− 1.20	− 1.22, 0.30	0.231	–	–	–	**–**
**Religion**										
Not religious	104	27.3	Ref	–	–	**–**	Ref	–	–	–
Religious	277	72.7	0.11	2.22	0.07, 1.09	**0.027**	0.05	0.93	−	0.353^a^
**Psychological trauma**										
No	206	54.1	Ref	–	–	–	Ref	–	–	–
Yes	175	45.9	0.20	3.87	0.44, 1.35	** < 0.001**	0.11	2.20	0.05, 0.94	**0.028**
**Smoking**										
No	294	77.2	Ref	–	**–**	*–*	Ref	*–*	***–***	*–*
Yes	87	22.8	− 0.144	− 2.83	− 1.32, − 0.24	**0.005**	− 0.12	− 2.50	− 1.15, − 0.14	**0.013**
**Drinking (≥ 3 times per week)**										
No	366	96.1	Ref	–	**–**	*–*	–	–	***–***	*–*
Yes	15	3.9	− 0.0005	− 0.01	− 1.19, − 1.18	0.993	–	–	–	**–**
**Exercise (≥ 3 days per week)**										
No	129	33.9	Ref	–	**–**	*–*	–	–	***–***	*–*
Yes	252	66.1	− 0.01	− 0.25	− 0.55, 0.42	0.801	–	–	–	–
**Regular diets (≥ 5 days per week)**										
No	38	10.0	Ref	–	–	**–**	–	–	–	–
Yes	343	90.0	0.002	0.04	− 0.75, 0.78	0.968	–	–	–	–
**Chronic disease (medical)**										
No	217	57.0	Ref	–	–	**–**	Ref	–	–	–
Yes	164	43.0	0.18	3.58	0.38, 1.29	** < 0.001**	0.12	2.55	0.13, 0.998	**0.011**

β: standardized coefficients; t: T score; CI: Confidence interval; SD: Standard deviation; **Bolds:** *p* < 0.005; ^a^: excluded from stepwise regression.

### Predictors for the level of sleep disturbance

The results of the linear regression are presented in Table 1. The results of the univariate linear regression revealed that higher levels of sleep disturbance were significantly associated with higher levels of social distance (standardized coefficients β = 0.11; *P* = 0.031), higher levels of social anxiety (β = 0.36; *P* < 0.001), higher levels of social information (β = 0.18; *P* = 0.001), higher levels of social adaptation (β = 0.14; *P* = 0.001), female subjects (β = 0.16; *P* = 0.002), subjects with religion (β = 0.11; *P* = 0.027), subjects with a history of psychological trauma (β = 0.20; *P* < 0.001), chronic disease (β = 0.18; *P* < 0.001), and those who did not smoke (β = − 0.14; *P* = 0.005).

The stepwise linear regression excluded level of social distance, social information, social adaptation, female, and religion subjects. The final model for the significant factors presented as follows: higher level of social anxiety (β = 0.30; *P* < 0.001), history of psychological trauma (β = 0.11; *P* = 0.028), chronic disease (β = 0.12; *P* = 0.011), and those who did not smoke (β = − 0.12; *P* = 0.013). The multivariate linear regression with 1000 bootstrapping methods demonstrated the same pattern for the independent associators for the level of sleep disturbance (Supplementary Table S5).

### Predictors for the level of COVID-19 related psychological distress

In Table 2, the results of the univariate linear regression revealed that higher levels of social distance (β = 0.16; *P* = 0.002), higher levels of social anxiety (β = 0.40; *P* < 0.001), higher levels of social information (β = 0.29; *P* < 0.001), higher levels of social adaptation (β = 0.19; *P* < 0.001), higher educational level (β = 0.15; *P* = 0.004), subjects without regular diets (β = − 0.14; *P* = 0.006), subjects with a history of psychological trauma (β = 0.15; *P* = 0.004), chronic disease (β = 0.11; *P* = 0.036), and those who did not smoke (β = − 0.12; *P* = 0.038), were significantly associated with psychological distress.

**Table 2:** Predictors for COVID-related psychological distress examined by univariate and stepwise multivariate linear regression (N = 381).

Predictors			Univariate regression				Stepwise multivariate regression			
	Mean	SD	β	t	95% CI	*p*	β	t	95% CI	*p*
Age (years)	50.43	11.34	− 0.03	− 0.55	− 0.03, 0.02	0.584	–	–	–	**–**
Education level (years)	12.02	3.20	0.15	2.88	0.04, 0.20	**0.004**	0.07	1.36	–	0.173^a^
Social distance	9.31	4.13	0.16	3.19	0.04, 0.16	**0.002**	− 0.06	0.99	–	0.324^a^
Social anxiety	3.48	1.75	0.40	8.57	0.46, 0.73	** < 0.001**	0.35	6.23	0.35, 0.67	** < 0.001**
Social information	4.24	1.90	0.29	5.86	0.26, 0.52	** < 0.001**	0.11	1.98	0.001, 0.30	**0.048**
Social adaptation	4.37	2.23	0.19	3.73	0.10, 0.33	** < 0.001**	− 0.03	1.06	–	0.637^a^

	*n*	%	β	t	95% CI	p	β	t	95% CI	p
**Sex**										
Male	232	60.9	Ref	–	**–**	–	–	–	**–**	–
Female	149	39.1	0.05	0.95	− 0.27, 0.78	0.344	–	–	–	–
**Occupational status**										
Employment	48	12.6	Ref	–	**–**	–	–	–	**–**	–
Unemployment	333	87.4	− 0.07	− 0.13	− 1.29, 0.27	0.195	–	–	–	**–**
**Marital status**										
Without partner	342	89.8	Ref	–	**–**	*–*	–	–	***–***	*–*
With partner	39	10.2	− 0.02	− 0.39	− 1.02, 0.68	0.695	–	–	–	**–**
**Religion**										
Not religious	104	27.3	Ref	–	–	**–**	**–**	**–**	**–**	**–**
Religious	277	72.7	0.08	1.54	− 0.13, 1.03	0.125	–	–	–	**–**
**Psychological trauma**										
No	206	54.1	Ref	–	–	–	Ref	–	–	–
Yes	175	45.9	0.15	2.91	0.25, 1.27	**0.004**	0.06	1.17	–	0.061^a^
**Smoking**										
No	294	77.2	Ref	–	**–**	*–*	Ref	–	***–***	*–*
Yes	87	22.8	− 0.12	− 2.08	− 1.26, − 0.03	**0.038**	− 0.07	− 1.40	–	0.161^a^
**Drinking (≥ 3 times per week)**										
No	366	96.1	Ref	–	**–**	*–*	–	–	***–***	*–*
Yes	15	3.9	0.004	0.08	− 1.28, 1.38	0.936	–	–	–	**–**
**Exercise (≥ 3 days per week)**										
No	129	33.9	Ref	–	–	*–*	–	–	***–***	*–*
Yes	252	66.1	0.08	1.49	− 0.13 0.96	0.137	–	–	–	–
**Regular diets (≥ 5 days per week)**										
No	38	10.0	Ref	–	**–**	*–*	Ref	–	–	–
Yes	343	90.0	− 0.14	− 2.78	− 2.06, − 0.35	**0.006**	− 0.15	− 3.24	− 2.07, − 0.51	**0.001**
**Chronic disease (medical)**										
No	217	57.0	Ref	–	**–**	*–*	Ref	–	–	–
Yes	164	43.0	0.11	2.11	0.04, 1.08	**0.036**	0.05	1.06	–	0.290^a^

β: standardized coefficients; t: T score; CI: Confidence interval; SD: Standard deviation; **Bolds:** *p* < 0.005; ^a^: excluded from stepwise regression.

Moreover, the stepwise linear regression excluded education level, level of social distance, social adaptation, history of psychological trauma, chronic disease, and those who did not smoke. The final model indicated that the following factors were considered significant: social anxiety (β = 0.35; *P* < 0.001), social information (β = 0.11; *P* = 0.048) and subjects without regular diets (β = − 0.15; *P* = 0.001). Multivariate linear regression with 1000 bootstrapping samples was conducted to verify the non-normality of the samples. However, level of social information was excluded from the bootstrapping analysis. The remaining factors that were examined by bootstrapping regression were social anxiety and subjects without regular diets (Supplementary Table S6).

### Tests for the mediation model and estimated co-efficient paths

**Figure 1:**
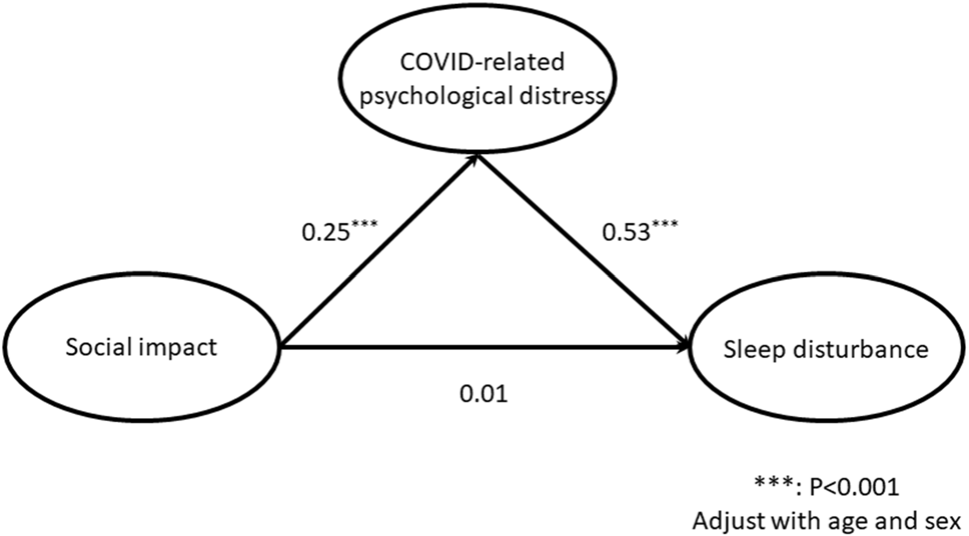
Final model of the mediating effect indicating the estimated coefficients of the paths.

The correlation matrix among variables is demonstrated in Table 3. In addition, the Table 4 showed the factor loadings through confirmatory factor analysis (CFA) and the result of the reliability test, which indicated an acceptable range. The indirect and direct effects were tested through the mediator mode after adjusting for age and sex, and the estimated path co-efficient is visualized in Fig. 1. According to the product terms of the path from social impact to COVID-19 related psychological distress (β = 0.25, *p* < 0.001) and the path from COVID-19 related psychological distress to sleep disturbance (β = 0.53, *p* < 0.001), the indirect effect at a value of 0.13 reached statistical significance (Sobel test: Z = 3.39; *P* < 0.05). In addition, the direct effect from social impact to sleep disturbance was not statistically significant. These results confirm the mediating effect of COVID-19 related psychological distress on the association between social impact and sleep disturbance. Based on the model fit index, we found that the hypothesized model had an adequate model fit index for χ2/df (2.34), GFI (0.948), AGFI (0.919), IFI (0.955), CFI (0.954), TLI (0.938), NFI (0.923), RMSEA (0.059), and SRMR (0.053), indicating that the hypothesized mediation model was a good-fitting model.

**Table 3:** The correlation matrix of observed variables.

	Mean	SD	2	3	4	5	6	7	8	9	10	11
1	1.37	0.79	0.64*	0.58*	0.59*	0.31*	0.32*	0.20*	0.29*	0.12*	0.27*	0.17*
2	1.25	0.66	–	0.67*	0.63*	0.36*	0.36*	0.17*	0.33*	0.16*	0.21*	0.13*
3	1.35	0.82		–	0.59*	0.31*	0.33*	0.16*	0.30*	0.098	0.26*	0.19*
4	1.31	0.78			–	0.35*	0.33*	0.18*	0.38*	0.15*	0.22*	0.14*
5	1.42	0.86				–	0.59*	0.46*	0.46*	0.08	0.11*	0.07
6	1.34	0.78					–	0.42*	0.40*	0.02	0.12*	0.04
7	1.55	0.65						–	0.53*	0.09	0.12*	0.14*
8	1.36	0.63							–	0.17*	0.22*	0.23*
9	9.31	4.13								–	0.54*	0.70*
10	4.24	1.90									–	0.49*
11	4.37	2.23										–

*: P < 0.05; 1 = DP-1; 2 = DP-2; 3 = DP-3; 4 = DP-4; 5 = Sleep -1; 6 = Sleep -2; 7 = Sleep -3; 8 = Sleep -4; 9 = SISQ-1; 10 = SISQ-2; 11 = SISQ-3.

**Table 4:** Principle component analysis for factors in the conceptual model.

Latent variables / Observed variables	Factor loading	Cronbach’s Alpha
**COVID-related psychological distress**		0.86
Hypervigilance or difficulty to be relaxed (DP-1)	0.76	
Emergence of somatic symptoms (DP-2)	0.84	
Efforts to avoidance (DP-3)	0.78	
Re-experience (DP-4)	0.76	
**Sleep disturbance**		0.78
Difficult to get to sleep (Sleep-1)	0.77	
Early wake up (Sleep-2)	0.71	
Subjective sleep quality (Sleep-3)	0.65	
Lack of enthusiasm (Sleep-4)	0.62	
**Societal Influences Survey Questionnaires (SISQ)**		0.75
Social distance (SISQ-1)	0.86	
Social information (SISQ-2)	0.63	
Social adaptation (SISQ-3)	0.80

## Discussion

To the best of our knowledge, this is the first study to comprehensively identify the predictors of the level of COVID-related psychological distress and sleep disturbance among patients with schizophrenia, as well as the first to develop a conceptual model to confirm the mediating effect of COVID-related psychological distress. After verifying the findings by stepwise and bootstrap regression, the present study reported that a higher level of COVID-related psychological distress was associated with a higher level of social anxiety and subjects without a regular diet. Sleep disturbance was associated with a higher level of social anxiety, a history of psychological trauma, chronic disease, and those who did not smoke.

In addition, it was revealed in the conceptual model that a higher level of social impact was indirectly associated with a higher level of sleep disturbance, and that this was positively mediated by the level of COVID-related psychological distress. Whereas, the direct effect from social impact to sleep disturbance did not reach statistical significance. The goodness-of-fit of multiple indices indicated the adequacy and applicability of the conceptual model.

Several factors of social impacts were observed to be associated with sleep disturbance and COVID-related psychological distress. When confronted with the uncertainty of COVID-19, people may suffer from mental health problems, such as anxiety^25^. It has been reported that the incidence rates of anxiety were up to 36% among inpatients with mental disorders during the COVID-19 outbreak^26^. Another case report reported that COVID-19-related anxiety can trigger a relapse of symptoms in patients with schizophrenia^27^. The current study further explored this hypothesis using quantitative analysis and demonstrated a significant association between anxiety over COVID-19 and COVID-related psychological distress, as well as sleep disturbance. Higher levels of anxiety were associated with more severe positive symptoms and poor function in subjects with schizophrenia^28^. Therefore, early assessment of anxiety levels due to COVID-19 in patients with schizophrenia is important to identify any undesirable impacts it could have on their mental status.

The current study revealed that social information is potentially associated with COVID-related psychological distress, even though it has been shown to be a protective factor that can help people cope with COVID-19 (by obtaining knowledge)^25^. Nevertheless, such behaviors may be associated with psychological burden. For example, over-exposure to COVID-related news has been shown to exacerbate anxiety and psychological distress among the general public^29^. In addition to enhancing well-adaptive coping with COVID-19, psychological interventions are also crucial for relieving distress. Higher levels of social distancing are also potentially associated with sleep disturbance and psychological distress. During the lockdown in Italy, mothers and their children reported adverse psychological difficulties, including sleep disturbance and emotional symptoms^30^. A British online survey reported that social distancing policies caused a significant negative impact on mental health and well-being, such as demotivation and decreased self-worth^31^. This increased stress due to isolation and even motivated people against social distancing^32^.

A history of psychological trauma was associated with a higher level of sleep disturbance. Individuals with previous traumatic events may suffer from nightmares, resulting in sleep disturbance^33^. The current study confirms this association among patients with schizophrenia during the COVID-19 pandemic. Moreover, healthcare workers should pay particular attention to the possible emergence of PTSD in the remission stage of COVID-19 for those who faced major psychological trauma, such as survival from a critical COVID-19 condition. Patients without a regular diet were shown to be associated with higher levels of COVID-related psychological distress, and this is comparable with previous studies. A previous review confirmed an association between healthy dietary habits and lower levels of depression or better mental health^34^. Another study revealed that psychological distress is associated with inadequate dietary intake^35^.

Higher levels of sleep disturbance were also associated with a history of chronic medical disease. Previous studies have demonstrated an association between sleep disturbance and medical status, such as heart^36^ or kidney disease^37^. It is especially important that this is taken note of by healthcare workers due to the higher degree of medical comorbidities among patients with schizophrenia^38^. Finally, it was a surprise that the current study revealed that smokers had lower levels of sleep disturbance than non-smokers. Individuals who smoke often suffer from sleep problems compared with non-smokers according to previous evidence^39^. Two different reasons could explain this finding. Firstly, some of the patients could be being treated with nicotine replacement therapy or varenicline, which may be associated with sleep problems^40^. However, information regarding status of treatment was not listed in the questionnaires. Secondly, some patients may smoke in an attempt to alleviate the undesirable side effects of antipsychotics, such as extrapyramidal symptoms^41^. Such symptoms are reported to be associated with sleep disturbance^42^.

Since the associations between social impact and sleep disturbance or COVID-related psychological distress were preliminarily identified and discussed above, the detailed etiology of the association remains unclear. Individuals may be hesitant to follow the instructions for active coping with COVID-19 if it is directly associated with sleep disturbance. Therefore, the conceptual model was built to clarify the direct effects of social impact on sleep disturbance, or to determine if there were any other factors mediating the association between social impact and sleep disturbance. The final model determined the key role of COVID-related psychological distress, and it revealed that higher levels of PTSD symptoms exaggerated sleep disturbance. About 70–91% of patients with PTSD have difficulty falling or staying asleep, and depending on the severity of their PTSD they can often complain of nightmares^43^. PTSD has been reported to be associated with shorter average duration and more episodes of rapid eye movement sleep^44^. Therefore, assessment and intervention for COVID-related psychological distress is beneficial to weaken the association between social impact and sleep disturbance for patients with schizophrenia.

The present study had several limitations that need to be stated. Firstly, the diagnosis of patients was through self-reported questionnaires. Since participants were either hospitalized, reside in an affiliated institution, or were being trained at the community rehabilitation center, no verification by psychiatrists through a diagnostic interview limited the interpretation of the current study. Second, the severity of schizophrenia was not estimated by research psychiatrists, which might be a confounding factor for the scoring of sleep disturbance. However, all of the included participants could realize the object of the study and followed instructions from the assistants. In addition, participants were not recruited from the acute ward, indicating that the severity of schizophrenia may not be so severe as to confound the findings of the current study. Finally, a single-center study may limit the generalizability and applicability of the results to other populations.

Several predictors were identified for the level of sleep disturbance and COVID-related psychological distress among patients with schizophrenia. The current study demonstrated the importance of COVID-related psychological distress. Patients with schizophrenia may suffer from sleep disturbance if they follow the instructions from the government to maintain social distancing and to gathering additional information about COVID-19. Since COVID-related psychological distress plays a key role in the association between social impacts and sleep disturbance, it is supposed that timely intervention for psychological distress may help patients with schizophrenia diminish the level of sleep disturbance they experience and could even improve their adherence to infection control policies. Regular screening and prompt treatment of COVID-related psychological distress and related burdens will be beneficial for patients with schizophrenia during the COVID-19 pandemic. In addition, healthcare workers should pay close attention to patients with a history of psychological trauma and physical comorbidities, as they have a significant association with sleep disturbance and COVID-related psychological distress. To be more specific to the threat of COVID-19, regular intervention to mental health problems should be adjusted. For instance, cognitive behavioral therapy could be effective within patients with schizophrenia as well as sleep disturbance^45^. However, threats of COVID-19 may compromise the efficacy, such as block down. Therefore, alternative intervention should be developed, such as teletherapy or digital health intervention^4^. Adjustment of frequency for follow up at outpatient department and programs to supply essential needs for patients with social-economic dysfunction are also crucial for patients with schizophrenia. Further studies are warranted to help extend the findings of the present study. For instance, a study with longitudinal follow-up could be helpful to better understand the psychological and social impact on patients at different stage of the pandemic.

## Methods

### Participants and ethics

Participant data for the current study was derived from a project that assessed the psychological and social impact of COVID-19 on medical staff, patients with a mental disorder, and their family at Kaohsiung Municipal Kai-Syuan Psychiatric Hospital (KSPH), where is located in southern-western Taiwan, and affiliated institutes (nursing home, half-way house, and community rehabilitation center). Printed advertisements were posted in the public area of the hospital and affiliated institutes to recruit participants. The recruiting period was May 9th 2020 to May 31st 2020. It was a cross-sectional study using paper-and-pencil questionnaires, and research assistants who individually explained the procedures to the participants to help them complete the research questionnaires. A total of 1559 subjects participated in the project and the current study includes those who presented as having chronic schizophrenia. The inclusion criteria of the current study were: (1) participants who self-reported themselves as having schizophrenia and confirmed by medical records, (2) those identified as being chronically ill with schizophrenia from medical records or at least in partial remission (admitted in the chronic ward, day-care ward, half-way house, or nursing home of KSPH, or received training programs at the community rehabilitation center), (3) could understand the objective of the current study and follow the instructions, (4) aged between 20 and 80 years, and (5) gave informed consent. Data with missing values or from those who could not complete the questionnaire were excluded. The current study was approved by the Institutional Review Board of KSPH (approval no. KSPH-2020-03). It was conducted according to the current revision of the Declaration of Helsinki and Taiwanese national legal requirements (Human Subjects Research Act, Taiwan).

### Measures

#### COVID-related psychological distress from disaster-related psychological screening tests

The level of COVID-related psychological distress was measured using four questions according to the disaster-related psychological screening test (DRPST). The DRPST is a reliable and validated tool used to rapidly screen for major depressive disorders or post-traumatic stress disorder (PTSD) after a disaster^46,47^. Four questions were selected from the DRPST to measure the status of hypervigilance, somatic symptoms, avoidance, and re-experience of COVID-19, which had persisted for more than one week in the past month. Each item was rated on a five-point Likert scale, with scores ranging from 1 (not at all) to 5 (extreme). Higher total scores indicated higher levels of PTSD symptoms. The details of the questionnaires are listed in Supplementary Table S7.

### Sleep disturbance scales from the Pittsburgh Sleep Quality Index

The Pittsburgh Sleep Quality Index (PSQI) was developed to estimate sleep quality with adequate validity and reliability^48^. Four questions were selected from the PSQI to measure the level of sleep disturbance, including difficulty in getting to sleep, ease of waking up during the night, subjective sleep quality, and enthusiasm during the past month (Supplementary Table S7). Each item was evaluated on a 4-point Likert scale, with scores ranging from 1 to 4. Higher total scores for the four items indicated a higher level of sleep disturbance.

### Social impact from the Societal Influences Survey Questionnaire

The Societal Influences Survey Questionnaire (SISQ) was developed to measure the psychological and social impact of the COVID-19 pandemic on individuals, and it was verified as having acceptable validity and reliability^25^. Ten of the questions were selected for the current study under four domains, including social distancing (compliance to keep social distance), social anxiety (anxiety due to COVID-19), social information (desire to seek related information), and social adaptation (awareness of progress of the pandemic overseas). Each question was composed of a 4-point Likert scale, with scores ranging from 1 (never) to 4 (often). Total scores for the above four domains were included into the analysis (Supplementary Table S7).

### Demographic characteristics

Other demographic information was recorded as continuous variables, including the participants’ age and their education level (years). Categorical variables including sex, occupational status, marital status, religion, history of psychological trauma, smoking (yes or no), drinking (≥ 3 times per week or not), regular exercise habits (≥ 3 days per week or not), regular diet habits (three or four meals a day, ≥ 5 days per week or not), and a history of chronic medical diseases, were also recorded. The threshold of regular diet habits was defined according to the routine of meals supplement in our hospital. In addition, irregular diet habits, such as meal skipping, have been reported to be associated with burden on mental health, such as distress and emotional problems^49^. The chronic medical diseases were identified as common medial comorbidities in patients with schizophrenia, such as hypertension, dyslipidemia, and diabetes mellitus^50^. Other diseases included coronary artery disease, hepato-biliary disease, gastric disease, lung disease, cancer or others (Supplementary Table S4). History of psychological trauma contained various kinds of trauma, including child abuse, biological disaster (pandemic), accident, etc. (Supplementary Table S4). The Kaohsiung Gas Explosion is a local traumatizing event in 2014, resulting in mental health burden^51^.

### Statistical analysis

Three steps of statistical analysis were conducted to better understand the interaction between social impact, COVID-19 related psychological distress, and sleep disturbance. All of the following analyses were processed using SPSS and AMOS version 23.0 for Windows (SPSS Inc., Chicago, IL, USA). Firstly, a descriptive analysis was used to present the demographic variables with details. Marital status was transformed into a dichotomous variable of either with partner (married and cohabited) or without partner (single, widowed, and divorced). History of psychological trauma and chronic medical disease were transformed into dichotomous variables of yes or no. To preliminary estimate the reliability and validity across all of the questionnaires in the current study (DRPST, PSQI, and SISQ) with local language (Traditional Chinese), Cronbach’s α was simply applied to test the reliability of each scale containing more than two questions. In addition, exploratory factor analysis was performed to verify the validity. The Kaiser–Mayer–Olkin (KMO) measure of sampling adequacy and Bartlett test were used to estimate the adequacy. The data was suitable for factor analysis when the KMO value was more than 0.60 and significant statistics (*p* < 0.05) was estimated from Bartlett test^52^. Total variance explained was estimated to evaluate how well a relevant notion can be measured^53^. The threshold at 50 percent of the total variance is acceptable in the field of psycho-social science^24^.

Secondly, univariate and stepwise multivariate linear regression was performed to initially clarify if the four categories of SISQ (social impact) and other demographic factors were associated with the dependent variables, including level of sleep disturbance and COVID-19 related psychological distress. The alpha level was set at 0.05. Because the non-normally distributed samples were identified with the significance of the Kolmogorov–Smirnov test (*p* < 0.001), the bootstrapping multiple linear regression with 1000 bootstrap samples was used to verify the results from the stepwise multivariate linear regression. The number of bootstrap samples was set as 1000 to obtain sufficiently accurate 95-percent bootstrap percentiles^54^. The 95% of the confidence interval was used to ascertain the statistical significance of the bootstrapping method, which can qualify the stability of the regression coefficient and reduce the length of the confidence interval^55^.

**Figure 2:**
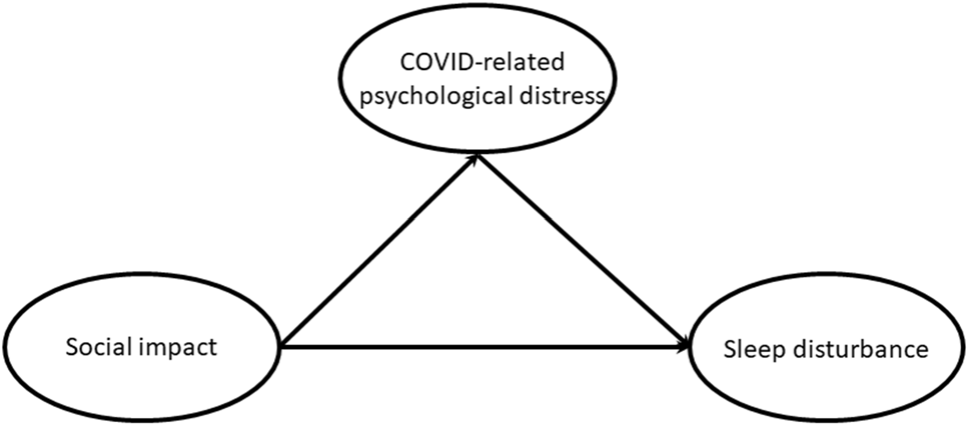
Conceptual model of the mediating effect.

Thirdly, if significant associations were identified between social impact, sleep disturbance, and COVID-19 related psychological distress, the conceptual model was further developed to test for an association between social impact and sleep disturbance, which was mediated by COVID-19 related psychological distress (Fig. 2). In this model there were eleven items composed of observed variables (indicators), including four questions on sleep disturbance, four questions on COVID-19 related psychological distress, and three total scores for dimensions (social distance, social information, and social adaptation) of social impacts. The total score for social anxiety was removed from the model because it measured a subjective level of anxiety. It may also have duplicated the score for COVID-19 related psychological distress. Three of the latent variables indicated social impact, COVID-19 related psychological distress, and sleep disturbance. The bivariate associations among the variables were estimated using Pearson’s correlation coefficient (*r*). Then, the two steps of structural equation modeling (SEM) were used. CFA was initially used to verify the association between latent variables and their indicators in the measurement model. Factor loading was applied as an index to assess the scale reliability between the indicators and the corresponding latent variables in the CFA. In addition, Cronbach’s α was reported to examine the internal consistency reliability. The range was considered acceptable if Cronbach’s α was > 0.5^23^.

The latent variable path analysis with maximum likelihood parameter estimations was then used to test the adequacy of the model and the direct/indirect effects of social impacts on sleep disturbance through COVID-19 related psychological distress^56^. As the Kolmogorov–Smirnov tests for sex and age were significant (*p* < 0.001; *p* = 0.001), indicating that there was non-normal distribution, age and sex were also included in this model as covariates to adjust for their effects on the latent variables. The standardized estimates (beta coefficient; β) were reported for the predictive strength explained in the model. The Sobel test was applied to examine the mediating effect^57^. In addition, multiple indices were applied to estimate the adequacy of the model. For each of these fit indices, the values indicating an acceptable model fit were as follows: Chi-Square goodness-of fit test (χ2/df < 5.0); Goodness of Fit Index (GFI ≥ 0.9); Adjusted Goodness of Fit Index (AGFI ≥ 0.9); Incremental Fit Index (IFI ≥ 0.95); Comparative Fit Index (CFI ≥ 0.95); Tucker-Lewis Index (TLI ≥ 0.9); Normed Fit Index (NFI ≥ 0.9); Root-Mean Square Error of Approximation (RMSEA < 0.08); and Standardized Root Mean Square Residual (SRMR ≤ 0.08)^58–60^.

## Supplementary Information

Supplementary Information.
