# Supplementary material for: COVID-related psychological distress fully mediates the association from social impact to sleep disturbance among patients with chronic schizophrenia
Source: Sci Rep. 2021 Aug 16;11:16524. doi: 10.1038/s41598-021-96022-2 (PMC8368012; doi:10.1038/s41598-021-96022-2)
Supplement: Supplementary file 1 — Supplementary Information. [file 41598_2021_96022_MOESM1_ESM.docx]

**COVID-related psychological distress fully mediates the association from social impact to sleep disturbance among patients with chronic schizophrenia**

Dian-Jeng Li; Li-Shiu Chou; Frank Huang-Chih Chou^*^; Su-Ting Hsu; Kuan-Ying Hsieh; Hui-Ching Wu; Wei-Tsung Kao; Guei-Ging Lin; Wei-Jen Chen; Joh-Jong Huang^*^

*contributed equally as corresponding authors

**Supplementary Table S1** Reliability and validity across the three questionnaires

| Variables | Cronbach’s Alpha | KMO value | Bartlett test ^a^ | Total variance explained |
| --- | --- | --- | --- | --- |
| COVID-related psychological distress | 0.86 | 0.828 | <0.001 | 61.774% |
| Hypervigilance or difficulty to be relaxed (DP-1) |  |  |  |  |
| Emergence of somatic symptoms (DP-2) |  |  |  |  |
| Efforts to avoidance (DP-3) |  |  |  |  |
| Re-experience (DP-4) |  |  |  |  |
| Sleep disturbance | 0.78 | 0.751 | <0.001 | 47.734% |
| Difficult to get to sleep (Sleep-1) |  |  |  |  |
| Early wake up (Sleep-2) |  |  |  |  |
| Subjective sleep quality (Sleep-3) |  |  |  |  |
| Lack of enthusiasm (Sleep-4) |  |  |  |  |
| Societal Influences Survey Questionnaires (SISQ) | 0.75 | 0.744 | <0.001 | 53.289% |
| Social distance (SISQ-1) |  |  |  |  |
| Social information (SISQ-2) |  |  |  |  |
| Social adaptation (SISQ-3) |  |  |  |  |

KMO value: Kaiser–Mayer–Olkin value; ^a^: significance of Bartlett test

**Supplementary Table S2** Distribution of marital status

| Marital status | n | % |
| --- | --- | --- |
| Single | 278 | 73.0 |
| Married | 39 | 10.3 |
| Divorced | 56 | 14.7 |
| Widowed | 7 | 1.8 |
| Cohabited | 1 | 0.3 |

**Supplementary Table S3** Distribution of events for psychological trauma

| Psychological trauma | n | % |
| --- | --- | --- |
| Biological disaster | 97 | 25.5 |
| Earthquake | 41 | 10.8 |
| Typhoon/ Flood/ Landslide | 32 | 8.4 |
| Kaohsiung Gas Explosion | 10 | 2.6 |
| Domestic violence in marriage | 25 | 6.6 |
| Victim of sexual assault | 7 | 1.8 |
| Childhood abuse | 16 | 4.2 |
| Unintentional accident | 11 | 2.9 |
| Military exercise/ war | 3 | 0.8 |
| Criminal events/ violence | 15 | 3.9 |
| Suicide/ self-harm | 26 | 6.8 |
| Others | 22 | 5.8 |

**Supplementary Table S4** Distribution of chronic disease (medical)

| Chronic medical disease | n | % |
| --- | --- | --- |
| Hypertension | 50 | 13.1 |
| Dyslipidemia | 29 | 7.6 |
| Diabetes mellitus | 41 | 10.8 |
| Coronary artery disease | 35 | 9.2 |
| Hepato-biliary disease | 14 | 3.7 |
| Gastric disease | 14 | 3.7 |
| Lung disease | 2 | 0.5 |
| Cancer | 2 | 0.5 |
| Others | 41 | 10.8 |

**Supplementary Table S5.** Predictors for level of sleep disturbance verified with multivariate linear regression with 1000 bootstrapping samples

| Predictors | β | 95% CI | p |
| --- | --- | --- | --- |
| Social distance | -0.19 | -0.09, 0.06 | 0.601^a^ |
| Social anxiety | 4.30 | 0.27, 0.61 | **0.001** |
| Social information | -0.04 | -0.18, 0.11 | 0.621^a^ |
| Social adaptation | -0.01 | -0.15, 0.12 | 0.655^a^ |
| Sex |  |  |  |
| Male | Ref | - | - |
| Female | 0.33 | -0.19, 0.82 | 0.200^a^ |
| Religion |  |  |  |
| Not religious | Ref | - | - |
| Religious | 0.19 | -0.24, 0.63 | 0.400^a^ |
| Smoking |  |  |  |
| No | Ref | - | - |
| Yes | -0.52 | -0.97, -0.08 | **0.023** |
| Psychological trauma |  |  |  |
| No | Ref | - | - |
| Yes | 0.48 | 0.07, 0.90 | **0.031** |
| Chronic disease (medical) |  |  |  |
| No | Ref | - | **-** |
| Yes | 0.54 | 0.10, 0.98 | **0.018** |

^a^: excluded from bootstrapping methods; **Bolds:** p<0.005

**Supplementary Table S6.** Predictors for level of COVID-related psychological distress verified with multivariate linear regression with 1000 bootstrapping samples

| Predictors | β | 95% CI | p |
| --- | --- | --- | --- |
| Social distance | -0.03 | -0.13, 0.06 | 0.514^a^ |
| Social anxiety | 0.49 | 0.30, 0.69 | **0.001** |
| Social information | 0.16 | -0.02, 0.344 | 0.089^a^ |
| Social adaptation | -0.01 | -0.18, 0.15 | 0.879^a^ |
| Education level |  |  |  |
| Smoking |  |  |  |
| No | Ref | - | - |
| Yes | -0.38 | -0.81, 0.10 | 0.101^a^ |
| Regular diets (≥5 days per week) |  |  |  |
| No | Ref | - | - |
| Yes | -1.30 | -2.62, -0.14 | **0.044** |
| Psychological trauma |  |  |  |
| No | Ref | - | **-** |
| Yes | 0.26 | -0.22, 0.75 | 0.305^a^ |
| Chronic disease (medical) |  |  |  |
| No | Ref | - | **-** |
| Yes | 0.26 | -0.20, 0.73 | 0.276^a^ |

^a^: excluded from bootstrapping methods; **Bolds:** p<0.005

**Supplementary Table S7** Measures used in this study

| Measures | Items | Response scale |
| --- | --- | --- |
| COVID-related psychological distress (DRPST ^1^): In recent one month, do you have the following symptoms persisted more than one week due to COVID-19? | Item 1: Hypervigilance or difficulty to be relaxed, even without information about COVID-19. | 1 = not at all, 2 = minimal, 3 = moderate, 4 = predominant, 5 = extreme |
|  | Item 2: Emergence of somatic symptoms (e.g. palpitation, tremor, sweating, or muscle rigidity) when hearing about COVID-19 | 1 = not at all, 2 = minimal, 3 = moderate, 4 = predominant, 5 = extreme |
|  | Item 3: Efforts to avoid activities, places, people or information that arouse recollections of the COVID-19. | 1 = not at all, 2 = minimal, 3 = moderate, 4 = predominant, 5 = extreme |
|  | Item 4: Acting or feeling as if the trauma were recurring (re-experience), and feel distressed. | 1 = not at all, 2 = minimal, 3 = moderate, 4 = predominant, 5 = extreme |
| Sleep disturbance (PSQI ^2^): During the past month,…. | Item 1: Do you cannot get to sleep within 30 minutes? | 1 = not during the past month, 2 = less than once a week, 3 = once or twice a week, 4 = three or more times a week. |
|  | Item 2: Do you wake up in the middle night or early morning, and you cannot sleep again? | 1 = not during the past month, 2 = less than once a week, 3 = once or twice a week, 4 = three or more times a week. |
|  | Item 3: How would you rate your sleep quality overall? | 1 = very good, 2 = fairly good, 3 = fairly bad, 4 = very bad. |
|  | Item 4: How much of a problem has it been for you to keep up enough enthusiasm to get things done? | 1 = not a problem at all, 2 = only a very slight problem, 3 = somewhat of a problem, 4 = a very big problem. |
| Social distance (SISQ ^3^): How has COVID-19 impacted you? | Item 1: I avoid communication with or encountering strangers. | 1 = never (0%), 2 = Rarely (10%), 3 = sometimes (30%), 4 = often (60% or above) |
|  | Item 2: I avoid close or personal contact with family members and/or people I am close to. | 1 = never (0%), 2 = Rarely (10%), 3 = sometimes (30%), 4 = often (60% or above) |
|  | Item 3: I avoid going out, especially if I should require public transport | 1 = never (0%), 2 = Rarely (10%), 3 = sometimes (30%), 4 = often (60% or above) |
|  | Item 4: I reduce eating out. | 1 = never (0%), 2 = Rarely (10%), 3 = sometimes (30%), 4 = often (60% or above) |
| Social anxiety (SISQ ^3^): How has COVID-19 impacted you? | Item 1: I worry about the pandemic affecting my work. | 1 = never (0%), 2 = Rarely (10%), 3 = sometimes (30%), 4 = often (60% or above) |
|  | Item 2: I feel anxious or fearful due to the pandemic. | 1 = never (0%), 2 = Rarely (10%), 3 = sometimes (30%), 4 = often (60% or above) |
| Social information (SISQ ^3^): How has COVID-19 impacted you? | Item 1: I constantly check for latest pandemic news updates via television, computer or phone | 1 = never (0%), 2 = Rarely (10%), 3 = sometimes (30%), 4 = often (60% or above) |
|  | Item 2: I continuously seek out information regarding COVID-19. | 1 = never (0%), 2 = Rarely (10%), 3 = sometimes (30%), 4 = often (60% or above) |
| Social adaptation (SISQ ^3^): How has COVID-19 impacted you? | Item 1: I am more cautious of residents from severely impacted areas | 1 = never (0%), 2 = Rarely (10%), 3 = sometimes (30%), 4 = often (60% or above) |
|  | Item 2: I avoid or cancel traveling overseas  (Answer “60%”, if you have cancelled an overseas trip. Answer “10%” or “30%”, if you are still considering your cancellation.) | 1 = never (0%), 2 = Rarely (10%), 3 = sometimes (30%), 4 = often (60% or above) |

^1^: DRPST= Disaster-Related Psychological Screening Test

^2^: PSQI= Pittsburgh Sleep Quality Index

^3^: SISQ= Societal Influences Survey Questionnaires
